# Transcatheter closure of patent ductus arteriosus: Evaluating the effect of the learning curve on the outcome

**DOI:** 10.4103/0974-2069.52804

**Published:** 2009

**Authors:** Ahmad S Azhar, Ayman A Abd El-Azim, Hamed S Habib

**Affiliations:** Department of Pediatric Cardiology, The International Medical Center, Jeddah, Saudi Arabia; 1 King Abdulaziz University, Jeddah, Saudi Arabia

**Keywords:** Amplatzer duct occluder, coil closure, complications, experience

## Abstract

**Background and Objectives:**

Initial experience with transcatheter closure of patent ductus arteriosus (PDA) using detachable coils and Amplatzer duct occluder devices is reported. We evaluated the outcome, complications, and influence of the learning curve, and also assessed the need of surgical backup for such interventional procedures.

**Methods:**

From January 2000 to December 2004, 121 patients underwent transcatheter closure of PDA. Aortic angiogram was performed to evaluate the size, position, and shape of the duct for appropriately choosing the occluder device type and size. A second aortic angiogram was performed 10 minutes after device deployment. Echocardiography was repeated at intervals of 24 hours, then at 1, 3, and 6 months after the procedure to assess complications. Stepwise multiple regression analysis was used to assess the role of experience in improving the outcome of the procedure.

**Results:**

Of 121 cases, four patients had pulmonary artery embolization of the occluder device which was successfully retrieved in the catheterization laboratory, while two others had embolization that required surgical intervention. Four patients had temporary residual leak, nine had protrusion of the device into the aorta without significant Doppler pressure gradient or hemolysis on follow-up, and five had partial hemodynamically insignificant obstruction to the left pulmonary artery. Statistical analysis showed that the effect of the learning curve and experience was responsible for 93% improvement in the procedural outcome over the five-year study period.

**Conclusion:**

Transcatheter occlusion of PDA is safe and effective alternative to surgery. Complications occurred in those with unfavorable duct anatomy and with the use of multiple coils. Surgical backup was important for such interventional procedures. Experience played a major role in the proper choice of device type and size which greatly influenced the outcome of the procedure.

## INTRODUCTION

Patent ductus arteriosus (PDA) accounts for 6-11% of all congenital heart defects.[1,2] Complications of PDA include congestive heart failure, repeated chest infections, pulmonary hypertension, and an increased risk of infective endocarditis. Transcatheter closure of PDA has largely replaced surgical ligation in different age groups.[3–7] Currently, surgical intervention is restricted to premature babies or small infants with large symptomatic PDA, cases with unfavorable duct anatomy, and whenever the cost of the closure devices is unaffordable.[1] Common complications of transcatheter closure of PDA include residual shunt,, left pulmonary artery (LPA) obstruction, protrusion of the device into the aorta, and embolization of the device[8–10] Incidence of complications increases with certain types and large size ducts, and with the use of multiple coils for occlusion.[11] There are only a few reports correlating outcome and complications with the learning curve and experience.[12–14] In this study, we are reporting our initial experience with PDA closure using detachable coils and Amplatzer duct occluder (ADO). Our focus was on reporting the complications of transcatheter closure of PDA using different PDA closure devices and comparing them with others. We also evaluated the influence of the learning curve and experience of the cardiologist on the outcome and complications.

## METHODS

The study was conducted at a moderate sized pediatric cardiac center, established in Al-Noor Hospital, Makkah, Saudi Arabia, in 1999. The study was approved by the Ethics committee of the hospital. From January 2000 to December 2004, 121 patients underwent transcatheter closure of PDA. A single, recently certified pediatric cardiologist performed all the procedures. Seventy six patients were females and 45 were males. Age ranged from 4 months to 18 years (median 5.1 years), weight from 4-55 kg (median 15.3 kg), PDA length was between 2-10 mm (median 6.2 mm), and the PDA diameter at the narrowest point ranged from 1-10 mm (median 2.9 mm). Fifteen patients had other associated lesions in the form of ventricular septal defect, atrial septal defect, and mild pulmonary valvular stenosis; however, none of these lesions required any treatment or intervention.

All patients had clinical evaluation and echocardiographic confirmation of the diagnosis. Cardiac catheterization was done for hemodynamic assessment and shunt estimation. This was followed by a biplane aortic angiogram to evaluate the size, position, and shape of the duct for appropriate choice of closure device type and size. Devices used for occlusion were detachable coils and ADOs.

Detachable coils were used for patients with small PDAs of ≤2.5 mm at the narrowest diameter. ADOs were used for PDAs that were >2.5 mm.[15] However, some exceptions to this rule had to be made due to nonavailability of the devices at the time of procedure. The sizes of the ADOs used were 2 mm larger than the narrowest diameter of the PDA. The length and diameter of the coils used were double the length and diameter of the PDA.

A second aortic angiogram was performed 10 minutes after device deployment. The decision regarding using multiple coils was made after documenting the residual shunt following the deployment of the first coil. Echocardiography was repeated at intervals of 24 hours and 1, 3, and 6 months after the procedure to assess residual leak and other complications.

Procedures lasted 45-180 minutes (median 115 minutes). Fluoroscopy time ranged between 5-36 minutes (median 17 minutes). Shunt calculations revealed Qp/Qs of 1.2-3.1 (median 2.1). The majority of patients (71%) had megaphone duct (type A). Table 1 summarizes the PDA types among the study population.

**Table 1:** Classification of patent ductus arteriosus types among patients

PDA type	Number of patients	Percentage
Megaphone (type A)	86	71
Window (type B)	0	0
Tubular (type C)	20	16.5
Aneurysmal (type D)	13	10.7
Conical (type E)	2	1.7

Detachable coils were used in 75 cases (62%), whereas ADOs were used in 46 patients (38%). Ninety three patients (77%) attended the 6-month follow-up; unfortunately, the remaining 23% were lost to follow-up.

Multiple variables were evaluated to assess their effect on the outcome of the procedure. The stepwise multiple regression analysis was used to reduce the independent variables to the minimum number of effective variables. The software used was the SPSS version 15(SPSS ver. 15). The dependent variable was the complications encountered. The independent variables were age and weight of the patient, length and diameter of the PDA, procedure time, and cardiologist' experience.

## RESULTS

One hundred and twenty one patients underwent transcatheter closure of PDA during the study period. Twenty six patients had complications. Of these, 13 patients underwent PDA closure with detachable coils and 13 cases had their PDAs closed with ADOs. The commonest complication was protrusion of the device into the aorta, occurring in three cases with coils and six cases with ADOs. Partial LPA obstruction complicated five cases of which three had ADOs; none of them had baseline LPA stenosis. None of the patients had major blood loss requiring blood transfusion. Temporary loss of peripheral pulse was noted in two cases with ADOs.

Device embolization occurred in six cases. Four coils were successfully retrieved in catheterization laboratory. One Amplatzer device and one coil needed surgical intervention for retrieval. Residual leak was encountered in four cases (three coils and one Amplatzer). Two of them resolved within 24 hours, and the other two cases resolved after one month. No delayed device migration, hemolysis, thromboembolic manifestations, wire fractures, arrhythmias, or deaths were encountered.

**Figure 1:**
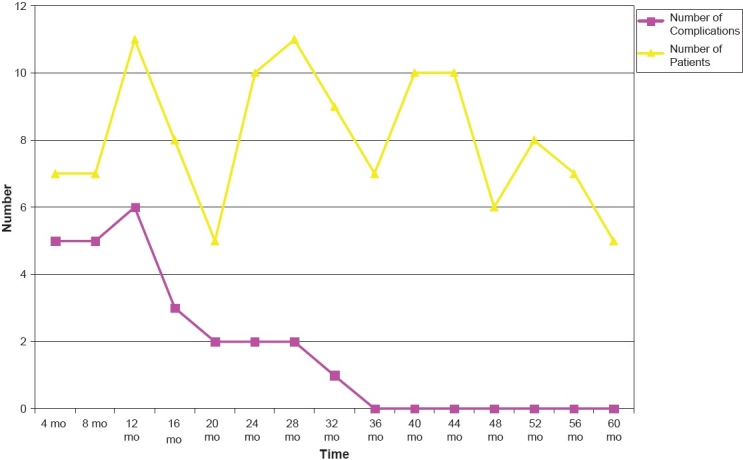
The declining incidence of complications during the five-year study period

Most complications occurred in the first two years of our experience after establishing the new interventional pediatric program. Incidence of complications was minimal in the remaining three years of the study period. Table 2 shows the incidence of complications throughout the five-year study period. Figure 1 shows the declining incidence of complications in relation to the number of patients over the five-year study period.

**Table 2:** Incidence of complications in the various periods of the 5-year experience

	1^st^ year	2^nd^ year	3^rd^ year	4^th^ year	5^th^ year
Residual leak	3	1	0	0	0
Device embolization	3	3	0	0	0
Protrusion into the aorta	5	2	2	0	0
LPA obstruction	3	1	1	0	0
Loss of peripheral pulses	2	0	0	0	0
Total number of complications	16	7	3	0	0
Number of patients	25	23	27	26	20
% of complications	64.00	30.35	11.11	0	0

Stepwise multiple regression analysis was performed to determine the effect of six independent factors (age and sex of patients, diameter and length of the duct, procedure time, and cardiologist's experience) on the occurrence of complications (the dependent factor). Two of the independent variables (experience and duct diameter) showed significant regression coefficients. The coefficient for experience was −0.23 indicating a significant negative relationship between experience and the occurrence of complications. On the other hand, duct diameter showed a coefficient of 0.56 indicating a significantly positive relationship with the incidence of the encountered complications. R square was 0.93 for the effect of experience alone, and 0.98 for the combined effect of experience and PDA diameter on the outcome.

## DISCUSSION

Transcatheter closure of PDA is a safe and effective alternative therapeutic modality to surgical intervention.[8,16–22] We report our experience with 121 cases performed over a period of five years with specific emphasis on complications and their relationship to cardiologist's experience.

Device embolization occurred in six cases (4.9%). Retrieval in the catheterization laboratory was successful in four cases. In all these four cases multiple coils were used for closure of large (≥4 mm diameter at the narrowest point) PDAs, and all coils embolized to the right pulmonary artery (RPA). Coils were used to close these large PDAs because ADOs were not available at the time of the procedure. In retrospect we believe that the cardiologist underestimated the size of the PDA and used smaller coils. Two out of the six cases required surgical intervention. In one of them, a large 8/4 coil embolized to the RPA and blocked a major branch and could not be retrieved in the catheterization laboratory. In the other case, a large 12/10 ADO embolized and obstructed the descending aorta in a patient with pulmonary hypertension, that is, right-to-left shunt. In this particular patient, the cardiologist elected to close the PDA because the patient had atrial septal defect, and it was thought that the pulmonary hypertension was reactive. All cases with embolization were within the first two years of our practice. This highlights the effect of the learning curve and the importance of experience in reducing the incidence of such a complication.[5,11,17] Only a few cases may require surgical intervention to retrieve embolized devices.[23] This occurred in two out of 121 cases (1.6%) in this study. Still, backup surgical service remains an important life-saving precaution. We believe it should be available in all interventional pediatric cardiac centers.

Protrusion of the occlusion device into the aorta is a common complication of transcatheter closure of PDA.[8–10] In our series, it occurred in nine cases (7%). In six of these cases ADO was used to close the PDA. The main reason was related to the size of the Amplatzer retention disc, which was larger than the size of the ductus ampulla. In one case, the duct was tubular (type C). In three cases, the ducts were of type A, but the coils used were larger in diameter than the ampulla. However, none of the cases had hemolysis or significant Doppler pressure gradient throughout the follow-up. The duct morphology plays a major role in the development of this particular complication; we observed that type C ducts and ducts with small ampulla are more prone to develop this complication. A specially designed device is recently used to reduce the incidence of device protrusion into the aorta.[24]

Obstruction to the LPA is another common complication of the procedure.[8–10] In our series, five cases (4%) developed this complication. Three of them had Amplatzer devices and two had coils. All five cases had short ductal length of <3 mm. None of them had clinical symptoms, and the highest pressure gradient measured by serial Doppler follow-up studies was 20 mm Hg.

Residual leak was noted in three cases with coils and only one case with Amplatzer device. Within 24 hours, it disappeared in two of them. At one-month follow-up, none had any residual leak. Thus, this particular complication was minimal in our series. Elimination of the residual leak is very important to prevent hemolysis.[25,26]

The results documented in our series are in line with the results reported in many other interventional pediatric cardiac centers around the world.[5,11,18,20,22,27–29] The complications encountered were mainly with large, short, and tubular (type C) ducts as has been reported earlier.[8,9,11,14,30,31]

Statistical analysis of the results showed a significant negative relationship between experience and complications. The effect of the learning curve was responsible for 93% improvement in the procedure outcome. Experience gained throughout the five-year study period was reflected on better case selection, accurate sizing of the duct length and diameter, better choice of closure device type and size, adopting different techniques to fit coils well in the ductus ampulla, and confirming the position of the device before releasing and deployment. This is highly indicative of the importance of experience and the rising learning curve in reducing the incidence of complications during transcatheter PDA closure.

## CONCLUSION

Our experience with transcatheter closure of PDA using detachable coils and AODs proved to be as safe and effective as reported in various interventional pediatric cardiology centers around the world. Residual leak was particularly minimal in our series. The results underline the importance of surgical backup to safeguard against certain complications. The learning curve and accumulating experience also play a major role in choosing the proper device and its size. This is crucial to minimize the complications.
